# Pectolinarigenin Induces Antioxidant Enzymes through Nrf2/ARE Pathway in HepG2 Cells

**DOI:** 10.3390/antiox11040675

**Published:** 2022-03-30

**Authors:** Mariko Shiraiwa, Tomoya Kitakaze, Yoko Yamashita, Yuichi Ukawa, Katsuyuki Mukai, Hitoshi Ashida

**Affiliations:** 1 Department of Agrobioscience, Graduate School of Agricultural Science, Kobe University, Nada-ku, Kobe 657-8501, Japan; mari.chapichapicon@gmail.com (M.S.); yoko.y@crystal.kobe-u.ac.jp (Y.Y.); 2 Graduate School of Life & Environmental Sciences, Osaka Prefecture University, Osaka 599-8531, Japan; kitakaze@biochem.osakafu-u.ac.jp; 3 Healthcare SBU Business Strategy, Daicel Corporation, Tokyo 108-8259, Japan; yi_ukawa@jp.daicel.com (Y.U.); mukai@gunma-u.ac.jp (K.M.)

**Keywords:** pectolinarigenin, antioxidant enzymes, nuclear factor-erythroid-2-related factor2

## Abstract

Pectolinarigenin (PG) and its glycoside pectolinarin (PN) were reported to have various health beneficial functions such as anti-inflammatory and anti-carcinogenic activities. It has also been reported that PG and PN have radical scavenging ability as direct antioxidant activity. However, the indirect antioxidant activity of PG and PN by inducing antioxidant enzymes in hepatocytes is not fully understood yet. In this study, we investigated whether PG and PN increase expression of antioxidant enzymes through the nuclear factor-erythroid-2-related factor 2 (Nrf2)-mediated pathway in human hepatoma HepG2 cells and the liver of male ICR mice. PG, but not PN, induced antioxidant enzymes, namely heme oxigenase-1, NAD(P)H:quinone oxidoreductase 1, and aldo-keto reductase family 1 member B10, in HepG2 cells. As for the induction mechanism of these enzymes, PG-induced nuclear accumulation of Nrf2 increased antioxidant response element (ARE)-mediated transcriptional activity and suppressed degradation of Nrf2 through modification of Kelch-like EXH-associated protein 1. Oral administration of PG also induced nuclear accumulation Nrf2 and expression of antioxidant enzymes in the liver of mice. Therefore, PG, but not PN, exhibits the indirect antioxidant activity by inducing antioxidant enzymes through the Nrf2/ARE pathway and may protect liver from oxidative stress.

## 1. Introduction

The liver plays an important role in the detoxification and metabolic conversion of exogenous chemicals and xenobiotics. Certain chemicals and xenobiotics cause oxidative stress during their detoxification and metabolic conversion processes. Oxidative stress is involved in the onset and progression of various liver diseases such as hepatitis C virus, hepatic cancer, and nonalcoholic fatty liver disease [1,2]. Certain flavonoids possess potent antioxidant activity based on the hydroxyl groups in their structures for scavenging singlet oxygen and various types of free radicals as the direct antioxidant activity [3,4].

In addition to the direct antioxidant activity, certain flavonoids are reported to induce antioxidant enzymes, such as heme oxigenase-1 (HO-1), NAD(P)H:quinone oxidoreductase 1 (NQO-1), and aldo-keto reductase family 1 member B10 (AKR1B10), mainly through phase 2 drug metabolism as an indirect antioxidant activity [5,6,7,8]. Induction of these enzymes is regulated at the transcriptional level by nuclear factor-erythroid-2-related factor 2 (Nrf2), a member of the NF-E2 family of the basic leucine zipper transcription factor. Under the normal conditions, Nrf2 constantly receives ubiquitination and proteasomal degradation by Kelch-like ECH-associated protein 1 (Keap1), a cytoplasmatic substrate adaptor protein for the Cullin3-containing E3-ligase complex. On the other hand, in the presence of reactive oxygen species (ROS) or electrophiles, Nrf2 detaches from Keap1 and stabilized Nrf2 translocates into the nucleus, where Nrf2 binds to antioxidant response element (ARE) and activates gene transcription for inducing antioxidant enzymes [9,10,11,12]. Therefore, the Keap1-Nrf2 system is recognized as one of the major cellular defense systems against oxidative stress by inducing antioxidant enzymes [13].

Pectolinarin (PN; 5-hydroxy-6-methoxy-2-(4-methoxyphenyl)-7-[(2*S*,3*R*,4*S*,5*S*,6*R*)-3,4,5-trihydroxy-6-[[(2*R*,3*R*,4*R*,5*R*,6*S*)-3,4,5-trihydroxy-6-methyl-tetrahydropyran-2-yl]oxymethyl]tetrahydropyran-2-yl]oxy-chromen-4-one, C_29_H_34_O_15_) and its aglycone pectolinarigenin (PG; 5,7-Dihydroxy-6-methoxy-2-(4-methoxyphenyl)chromen-4-one, C_17_H_14_O_6_) were identified as a major constituent in medicinal herbs, such as the Chinese herb *Cirsium chanroenicum* and the Korean herb *Cirsium setidens*. PN was found in 87 plants of 29 different genera and PG in 136 plants of 71 different genera [14], suggesting that certain plants contain aglycone PG but not PN. Both PN and PG have been reported to possess various health beneficial functions, such as anti-inflammatory [15], anti-carcinogenic [16], and anti-microbial [17] activities. These compounds are also reported to exhibit DPPH radical scavenging activity [18,19] and hepatoprotective activity against D-galactosamine-induced hepatic injury in rats [20]. However, the induction of antioxidant enzymes and its underlying molecular mechanism are not fully understood yet. In the present study, we investigated whether PN and PG induce antioxidant enzymes and the underlying induction mechanism by focusing on the Nrf2/ARE pathway.

## 2. Materials and Methods

### 2.1. Materials

**Figure 1:**
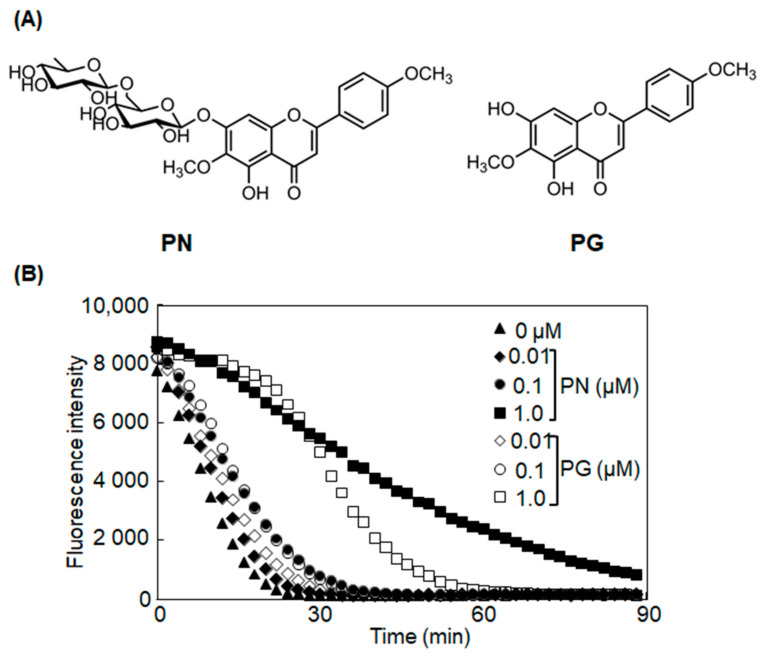
Reactive oxygen species (ROS) scavenging activity of Pectolinarin (PN) and Pectolinarigenin (PG) under cell-free conditions. (**A**) Molecular structures of PN and PG. (**B**) Fluorescence decay curve for antioxidant activity under cell-free conditions.

PN and PG were purchased from ChemFaces (Wuhan, China), and their chemical structures are shown in Figure 1A.; 2′,7′-Dichlorofluorescin diacetate (DCFH-DA) was obtained from Sigma-Aldrich (St. Louis, MO, USA); *tert*-Butylhydroquinone (*t*-BHQ), luteolin, and Immunostar^®^ LD chemiluminescence detection kit were purchased from FUJIFILM Wako Pure Chemical Co. (Osaka, Japan); Blocking One and Blocking One-P solutions were from Nacalai Tesque, Inc. (Kyoto, Japan); polyvinylidene difluoride membrane (PVDF) was from GE Healthcare (Fairfield, WA, USA); TRIzol, Lipofectamine 2000 reagent, and Lipofectamine^®^ RNAiMAX reagent were from Invitrogen (Waltham, MA, USA); RevaTra Ace was from Toyobo (Osaka, Japan); PicaGene Dual Sea Pansy Luminescence Kit was from TOYO INK (Tokyo, Japan). Antibodies against HO-1 were purchased from Enzo Life Sciences, Inc. (Farmingdale, NY, USA); NQO1 and phosphorylated Nrf2 (Ser40) were from Abcam (Cambridge, UK); AKR1B10 was from Abnova (Taipei, Taiwan); β-actin was from Cell Signaling Technology Co. (Denver, MA, USA); Nrf2 was from Medical & Biological Laboratories Co., Ltd., (Aichi, Japan); Keap1 and Lamin B were from Santa Cruz Biotechnology (Dallas, TX, USA); horseradish peroxidase (HRP)-conjugated antibodies against mouse IgG and goat IgG were purchased from Santa Cruz Biotechnology, and rabbit IgG was from Bio-Rad Laboratories Inc. (Hercules, CA, USA). All other regents used were of the highest grade available from commercial sources.

### 2.2. Antioxidant Activity under Cell-Free Conditions

Antioxidant activity of PN and PG under cell-free conditions was evaluated by measuring oxygen radical absorbing capacity using a 2,2′-azobis (2-amidinopropane) dihydrochloride (AAPH). Briefly, PN and PG at 0.01, 0.1, or 1.0 μM (final concentration) was added to a 96-well microplate followed by the addition of 70 nM fluorescein in 75 mM phosphate buffer (pH 7.4) and incubation for 15 min at 37 °C. As a positive control, Trolox, which is a cell-permeable and water-soluble analogue of vitamin E, was used. Then, 12 mM AAPH in 75 mM phosphate buffer (pH 7.4) was added to each well and fluorescence was recorded every 2 min for 120 min with the excitation wavelength and emission one at 485 nm and 535 nm, respectively, by a Wallac 1420 ARVOsx Multilabel Counter (Perkin-Elmer, Boston, MA, USA). Antioxidant activity was expressed as Trolox equivalent.

### 2.3. Cell Culture and Treatment

HepG2, human hepatoma cell line, was obtained from the American Type Culture Collection (Manassas, VA, USA). HepG2 cells were maintained and cultured in Dulbecco’s modified Eagle’s medium (DMEM, Nissui Pharmaceutical, Tokyo, Japan) containing 10% (*v/v*) fetal bovine serum (FBS; Sigma-Aldrich), 4 mM L-glutamine, 100 U/mL penicillin, and 100 µg/mL streptomycin under a humidified atmosphere of 95% (*v/v*) air and 5% (*v/v*) CO_2_ at 37 °C. After the cells were seeded on the culture dish or microplate and reaching 90% confluence, the cells were treated with various concentrations of PN and PG. DMSO (final concentration at 0.1%) was used as a vehicle control.

### 2.4. Cytotoxicity Assay

The cytotoxicity of PN and PG was evaluated by a crystal violet assay. HepG2 cells on the 96-wells plate were treated with PN and PG at 0.1, 1.0, and 10 μM for 24 h. The staining solution (0.2% Crystal violet in 2% ethanol) was added to each well for 10 min. After washing three times with tap water, the cells were solubilized in a mixture of 0.5% sodium dodecyl sulfate (SDS) in 50% ethanol. Absorbance of the solution was measured at 530 nm. Cell viability was shown as a percent of the control.

### 2.5. Measurement of an Intracellular ROS Level

To measure the intracellular ROS level in HepG2 cells, DCFH-DA, a cell- permeable fluorescent probe, was introduced. HepG2 cells on a 96-well plate were treated with PN and PG at 0.1, 1.0, and 10 μM for 1, 3, and 24 h. As a positive control, 30 μM *t*-BHQ was also treated to the cells. After the cells were washed with phosphate-buffered saline (PBS), 0.1 mM DCFH-DA was added to each well and incubated at 37 °C for 30 min. After the cells were washed again with PBS, 10 mM AAPH was added to each well and reacted for 30 min at room temperature. After the cells were washed twice with PBS, formed DCF from DCFH by ROS was determined by measuring fluorescence at excitation wavelength of 485 nm and emission one of 535 nm by an ARVO X4 plate reader (PerkinElmer, MA, USA). Data are represented as the fluorescence ratio of AAPH-treatment to the compound-treatment or non-treatment.

### 2.6. Animal Experiment

All animal experiments were approved by the Institutional Animal Care and Use Committee (The ethical protocol code: 2020-10-03, Permission date: 29 October 2020) and carried out according to the guidelines for animal experiments at Kobe University. Male ICR mice (6-week-old) were obtained from Japan SLC, Inc. (Shizuoka, Japan) and allowed free access to tap water and a purified diet AIN-93M (Research Diets, NJ, USA) in a temperature-controlled room (23 ± 2 °C) with 14:10 h light/dark cycle (lights on at 8:00 a.m.). The mice were randomly divided into five groups of six each and orally administrated PN and PG at 1.0 or 10 mg/kg body weight once a day for 1 week. For the control mice, polyethylene glycol as the vehicle control was administered at 5 mL/kg body weight. The mice were killed by exsanguination from cardiac puncture 2 h after the final administration. The plasma and liver were collected and frozen in liquid nitrogen and stored at −80 °C until analyzed.

### 2.7. Preparation of Lysate and Nuclear and Post-Nuclear Fractions

HepG2 cells and the liver of mice were homogenized with radioimmunoprecipitation assay (RIPA) buffer consisting of 10 mM Tris-HCl, pH 8.0, 150 mM NaCl, 1% (*v/v*) NP-40, 0.5% (*w/v*) deoxycholic acid, 0.1% (*w/v*) SDS, 1 mM dithiothreitol (DTT), and a cocktail of protease inhibitors (1 mM phenylmethylsulfonyl fluoride, 5 μg/mL aprotinin, and 5 μg/mL leupeptin). The homogenate was kept on ice for 1 h with occasional mixing and centrifuged at 12,000× *g* for 20 min at 4 °C. Obtained supernatant was used as lysate. For preparing the nuclear and post-nuclear fractions, the cells and liver were alternatively homogenized with buffer A consisting of 20 mM HEPES, pH 7.6, 20% (*v/v*) glycerol, 10 mM NaCl, 1.5 mM MgCl_2_, 0.2 mM EDTA, 1 mM DTT, 0.1% (*v/v*) NP-40, and the same protease inhibitor cocktail. The homogenate was centrifuged at 800× *g* for 10 min at 4 °C and the supernatant was used as a post-nuclear fraction. Precipitate was washed twice with buffer A under the same centrifugation conditions. Resultant precipitate was suspended in buffer B consisting of 20 mM HEPES, pH 7.6, 20% (*v/v*) glycerol, 500 mM NaCl, 1.5 mM MgCl_2_, 0.2 mM EDTA, 1 mM DTT, 0.1% (*v/v*) NP-40, and the same protease inhibitor cocktail. The mixture was kept on ice for 45 min with occasional tapping and centrifuged at 20,000× *g* for 20 min at 4 °C. Obtained supernatant was referred to a nuclear fraction.

### 2.8. Western Blotting

For the detection of HO-1, NQO1, AKR1B10, Nrf2, and Keap1, lysate was subjected to SDS-polyacrylamide gel electrophoresis. The proteins were transferred to PVDF. The membrane was incubated with commercially available blocking solution [Blocking One or Blocking one-P (for detection of phosphorylated proteins)] for 30 min at room temperature. The membrane was incubated with primary antibodies (1:5000) toward HO-1, NQO1, AKR1B10, Nrf2, pNrf2, Keap1, β-actin, and Lamin B overnight at 4 °C, followed by incubation with the corresponding HRP-conjugated secondary antibodies (1:50,000) toward rabbit IgG, mouse IgG, and goat IgG for 1 h at room temperature. The blots were developed using Immuno Star^®^ LD Western Blotting Substrate (Wako Pure Chemical). Specific band was detected with Light-Capture II (ATTO, Tokyo, Japan). The density of a specific band was quantified by ImageJ image analysis software (National Institutes of Health, Bethesda, MD, USA).

### 2.9. RNA Isolation and Quantitative Real-Time PCR

The total RNA was extracted from HepG2 cells using TRIzol (Invitrogen). cDNA was synthesized using RevaTra Ace (Toyobo) and subjected to quantitative real-time PCR (qPCR) using the following primers: *NFE2L2* (NM_006164: forward primer 5′-GACGGTATGCAACAGGACATTGAG-3′ and reverse one 5′-AACTTCTGTCAGTTTGGCTTCTGGA-3′) and *ACTB* (NM_001101: forward primer 5′-GGACTTCGAGCAAGAGATGG-3′ and reverse one 5′-AGCACTGTGTTGGCGTACAG-3′). *ACTB* mRNA was used as a normalized control. qPCR was performed by a two-step PCR method with SYBR PremixEx Taq II (Takara Bio., Kyoto, Japan).

### 2.10. Reporter Assay

An ARE-mediated transcriptional activity was measured according to our previous report [6]. Briefly, HepG2 cells were transfected with the following reporter vectors: pGL4.20-ARE-TATA-Luc and pRL-SV40 as a control reporter vector (Promega, Madison, WI, USA). After the medium was changed to a fresh one, PG was treated to the cells for 24 h. Transfection efficiency was normalized to that of pRL-SV40. The activities of Firefly and *Renilla* luciferase were measured by PicaGene Dual Sea Pansy Luminescence Kit (TOYO INK) and the ARVO X4 plate reader. Data are represented as the relative light units (ratio of the activity of *Renilla* luciferase to that of firefly one).

### 2.11. Transfection of Small Interfering RNA (siRNA)

HepG2 cells were seeded in a 24-well plate with antibiotic-free medium. Nrf2-specific siRNA (Thermo Fisher Scientific, Waltham, MA, USA) or control scrambled siRNA was transfected into the cells using Lipofectamine^®^ RNAiMAX reagent (Invitrogen) according to the manufacturer’s instructions. After 24 h, protein expression of Nrf2 in the cells was checked by western blotting and used for DCFH-DA assay after treatment with PG.

### 2.12. Quantitative Analysis of PG in HepG2 Cells and the Plasma of Mice

An incorporated amount of PG in HepG2 cells and the plasma of mice was quantified by high-performance liquid-chromatography (HPLC). After treatment with PN and PG at 10 µM to HepG2 cells for 3 and 24 h, the cells were homogenized with 1.0 mL of PBS containing the same cocktail of protease inhibitors as RIPA buffer by ultrasonication for 1 min. PN and PG was extracted with ethyl acetate from the cell homogenate according to the previous method [7]. On the other hand, the plasma was treated with or without deconjugation enzymes, glucuronidase, and sulfatase before the extraction with ethyl acetate. After the extraction, solvent was evaporated, residue was re-dissolved in 5 µL ethyl acetate and 45 µL acetonitrile containing 0.05% trifluoroacetic acid, then filtered through a 0.45 µm filter before being subjected to HPLC. The HPLC system consisted of a Shimadzu liquid chromatograph model CBM-20A (Kyoto, Japan) equipped with an autosampler using a Cadenza CL-C18 column (φ 250 mm × 4.6 mm, 3 μm, Imtakt, Kyoto, Japan) at a flow rate of 0.8 mL/min, column temperature of 40 °C, and UV detection at 330 nm. The mobile phase consisted of solvents A (0.05% trifluoroacetic acid in H_2_O) and solvents B (0.05% trifluoroacetic acid in acetonitrile). The gradient program was as follows: the initial composition consisted of 70% A and 30% B; followed by a linear gradient to 90% B over 15 min, 90% B hold for 5 min, then 30% B for 10 min [21].

### 2.13. Statistical Analysis

The data are expressed as the mean ± SE. Statistical significance was determined using Dunnett’s test, Tukey’s multiple-range test and student’s *t*-test as described in each figure legend using JMP statistical software version 11.2.0 (SAS Institute. Cary, NC, USA). The level of significance was set to *p <* 0.05.

## 3. Results

### 3.1. PN and PG Have Potent Antioxidant Activity under Cell-Free Conditions

We, first, confirmed the direct antioxidant activity of PN and PG by the oxygen radical absorbing capacity assay. Figure 1B shows the result of time- and concentration-dependent oxygen radical absorbing capacity of PN and PG by measuring the quenching of fluorescence. Both PN and PG at 1.0 µM retained the quenching of fluorescence, indicating that these compounds had the direct antioxidant activity. Although PN showed stronger than PG in comparison of the activity at the same molarity, oxygen radical absorbing capacity values, which were calculated from this result based on the weight of compound, were 3.9 × 10^3^ µmol TE/g for PN and 5.3 × 10^3^ µmol TE/g for PG, and the value per gram of PG was higher than that of PN.

Oxygen radical absorbing capacity of PN and PG was measured using 2,2′-Azobis (2-amidinopropane) dihydrochloride (AAPH) as described in the Section 2.2. fluorescence decay curve is shown in Figure 1B. Trolox was used as a positive control.

### 3.2. PG Inhibited AAPH-Induced ROS Accumulation in HepG2 Cells

**Figure 2:**
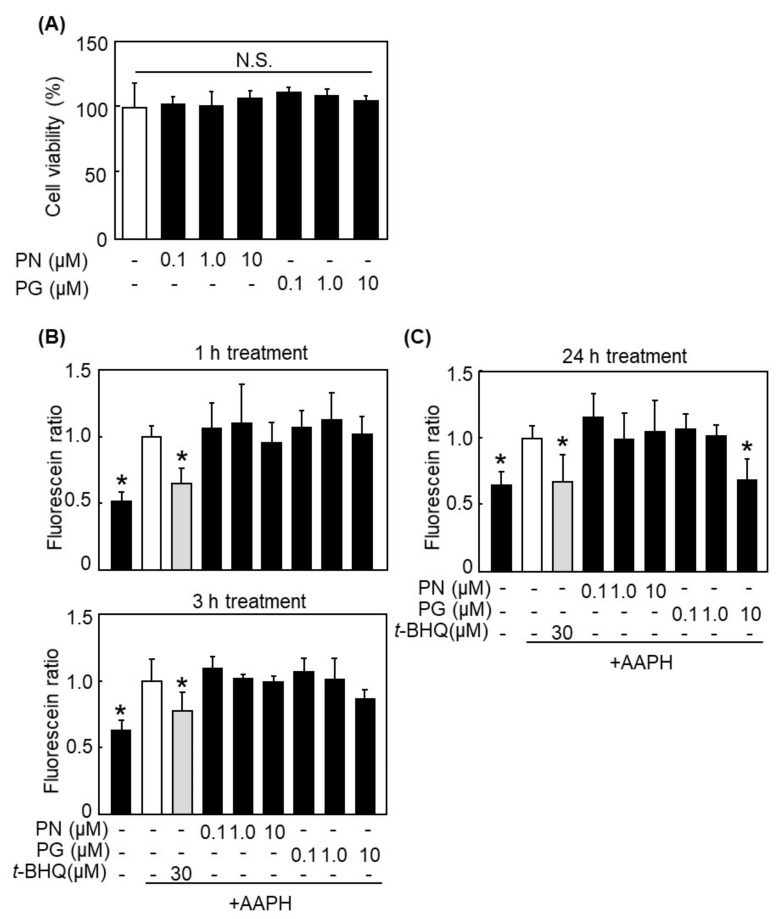
Suppression effect of PG on AAPH-induced ROS accumulation in HepG2 cells. (**A**) Cytotoxicity of PN and PG was evaluated by crystal violet assay as described in Section 2.4. (**B**,**C**) Effect of PN and PG on AAPH-induced ROS scavenging activity was evaluated by 2′,7′-Dichlorofluorescin diacetate (DCFH-DA) assay as described in Section 2.5. Phosphate-buffered saline (PBS) was used as a negative control instead of AAPH. *tertiary*-Butylhydroquinone (*t*-BHQ) was used as a positive control. Data are represented as the fluorescence ratio of AAPH-treatment to the compound-treatment or non-treatment. Values are the mean ± SE (*n* = 6), * *p* < 0.05 vs. control by Dunnett’s multiple comparison test. N.S.: Not significant.

Both PN and PG at 0.1, 1.0, and 10 μM did not show any cytotoxicity against HepG2 cells (Figure 2A). Under the same conditions, the inhibitory effect of PN and PG against the AAPH-induced intracellular ROS accumulation was evaluated using DCFH-DA. As shown in Figure 2B, neither PN nor PG decreased AAPH-induced ROS accumulation after 1- or 3 h-treatment, though PG at 10 µM slightly decreased the ROS accumulation after 3 h-treatment without statistical significance. On the contrary, PG at 10 µM significantly inhibited ROS accumulation after 24 h-treatment (Figure 2C). Incorporated PN and PG were determined by HPLC after 3 h- and 24 h-treatment with these compounds at 10 μM (20.0 nmols). After 3 h-treatment with PG to the cells, 17.2 ± 8.1 pmols of PG was detected in the cells. Intracellular concentration of PG was 0.34 ± 0.16 μM after calculation from the cell volume. However, PG was not detected after 24 h-treatment. PN was also not detected in both treatment times.

### 3.3. PG, but Not PN, Increased the Expression of Antioxidant Enzymes

**Figure 3:**
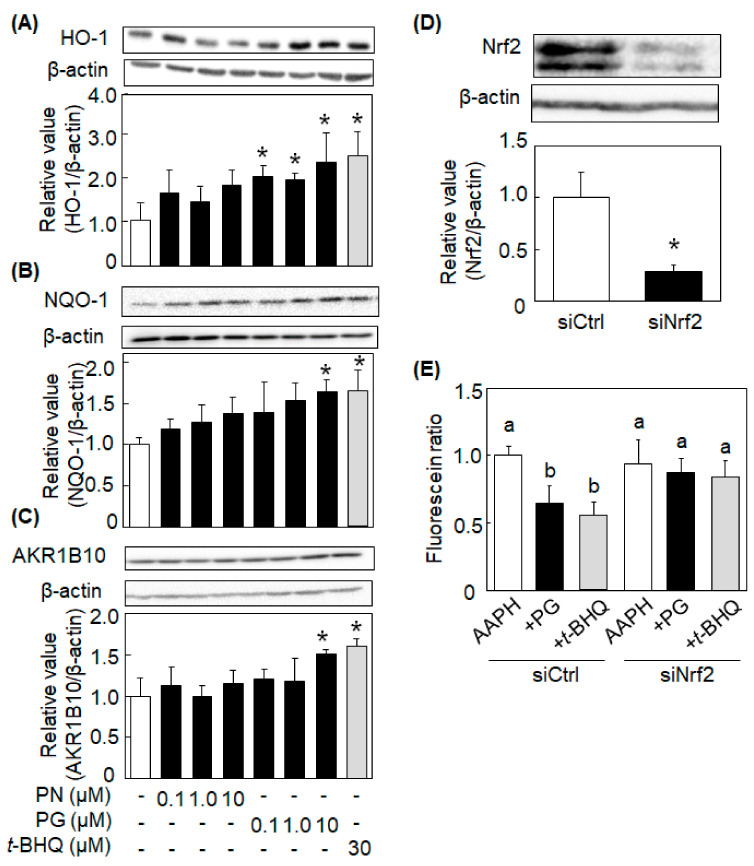
Induction of antioxidant enzymes by PG in HepG2 cells. HepG2 cells were treated with PN and PG and the protein expression level of antioxidant enzymes was detected by western blotting as described in Section 2.8. (**A**) Heme oxigenase-1 (HO-1), (**B**) NAD(P)H:Quinone oxidoreductase 1 (NQO1) and (**C**) Aldo-keto reductase family 1 member B10 (AKR1B10). Upper panels show typical blot data, while bottom ones are the intensity of specific bands after normalizing data by the β-actin level. Values are the mean ± SE (*n* = 3), * *p* < 0.05 vs. control by Dunnett’s multiple comparison test. (**D**) HepG2 cells were transfected with siRNA and down-expression of nuclear factor-erythroid-2-related factor 2 (Nrf2) was confirmed by western blotting. Values are the mean ± SE (*n* = 3), * *p* < 0.05 vs. control by Student’s *t*-test. (**E**) HepG2 cells were transfected with siNrf2, and AAPH-induced ROS scavenging activity was measured by DCFH-DA assay. Data are represented as the fluorescence ratio of AAPH alone to the compound-treatment. Values are the mean ± SE (*n* = 5). Statistical significance is indicated by different letters (*p* < 0.05) by Tukey’s multiple-range test.

Since PG decreased the ROS level after 24 h-treatment, the author speculated the antioxidant activity of PG was depending on the induction of antioxidant enzymes. After HepG2 cells were treated with PN and PG at 0.1, 1.0, and 10 μM for 24 h, expression of antioxidant enzymes was evaluated. PG at all doses used in this experiment significantly increased protein expression of HO-1 (Figure 3A). PG at 10 μM also significantly increased protein expression of NQO-1 and AKR1B10 (Figure 3B,C, respectively). On the other hand, PN had no effects on the expression of these enzymes. These results indicated that PG, but not PN, had the ability to induce antioxidant enzymes.

Since Nrf2 is known to regulate the induction of these antioxidant enzymes, it was investigated whether Nrf2 is involved in the PG-caused inhibition of ROS accumulation. To knockdown Nrf2 protein, siRNA was introduced and transfected to the cells. Significant decrease in the Nrf2 protein level was confirmed 24 h after treatment with siRNA or Nrf2 in HepG2 cells (Figure 3D). Under the same experimental conditions, PG- and *t*-BHQ-caused inhibition of the ROS accumulation was canceled by the treatment with siRNA for Nrf2, but not for control (Figure 3E). It is, therefore, suggested that Nrf2 is involved in the indirect antioxidant activity of PG through inducing antioxidant enzymes.

### 3.4. PG Activated the Nrf2/ARE Pathway

**Figure 4:**
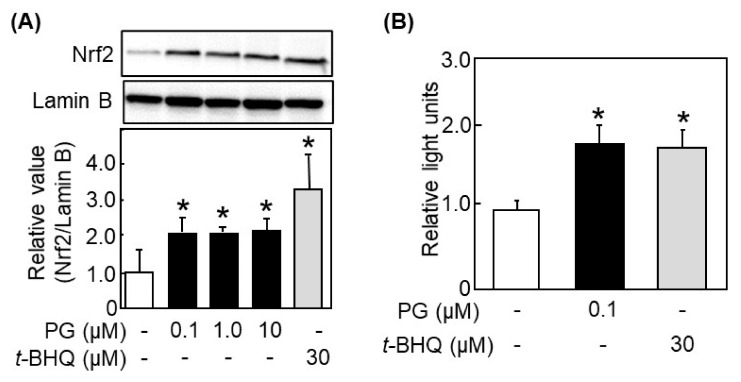
Nuclear accumulation of Nrf2 and ARE-mediated transcriptional activity in PG-treated HepG2 cells. (**A**) Nuclear accumulation of Nrf2 after treatment with PG to HepG2 cells was determined by western blotting. Th upper panel shows typical blot data, while the bottom one is the intensity of specific bands after normalizing data by the Lamin B level. (**B**) Effect of PG on ARE-mediated transcriptional activity was evaluated by Luciferase reporter assay as described in the Section 2.10. Values are the mean ± SE (*n* = 3), * *p* < 0.05 vs. control by Dunnett’s multiple comparison test.

Expression of HO-1, NQO-1, and AKR1B10 is regulated by binding of Nrf2 to the antioxidant response element (ARE) in the nucleus [9,10]. Kitakaze et al. reported that luteolin increased nuclear accumulation of Nrf2 after 3 h-treatment in HepG2 cells [6]. Thus, we also treated PG to HepG2 cells for 3 h and detected Nrf2 in nucleus. It was found that PG as well as *t*-BHQ significantly increased the nuclear accumulation of Nrf2 (Figure 4A). Next, ARE-mediated transcriptional activity was measured by the reporter gene assay. As a result, both PG and *t*-BHQ enhanced the ARE-mediated gene transcriptional activity (Figure 4B).

**Figure 5:**
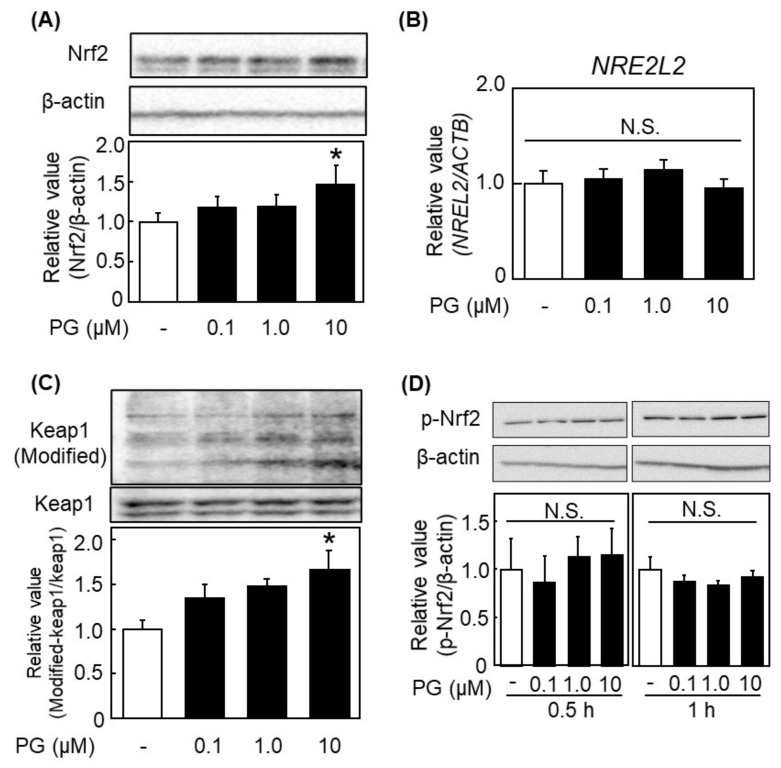
Stability of Nrf2 by inducing Keap1 modification in PG-treated HepG2 cells. After treatment with PG to HepG2 cells, expression of (**A**) Nrf2 and (**B**) its gene (*NFE2L2*) was measured by western blotting and qPCR, respectively. (**C**) Kelch-like ECH-associated protein 1 (Keap1) and its modification, and (**D**) phosphorylation of Nrf2 was determined by western blotting. after treatment with PG to the cells. (**A**,**C**,**D**), Upper panels show typical blot data, while bottom ones are the intensity of specific bands after normalizing data by the β-actin level. Values are the mean ± SE (*n* = 3), * *p* < 0.05 vs. control by Dunnett’s multiple comparison test. N.S.: Not significant.

When the expression of Nrf2 was examined, PG increased protein expression of Nrf2 (Figure 5A) without affecting its gene expression (Figure 5B). It is reported that certain compounds such as luteolin, *t*-BHQ, and sulforaphane induce modification of Keap1 to protect degradation of Nrf2 [6,22,23]. As shown in Figure 5C, PG caused modification of Keap1 and a significant effect was observed at 10 μM. Phosphorylation of Nrf2 at Ser 40 is also an important event for its activation [24], but PG did not cause the phosphorylation (Figure 5D). These results indicated that PG activated the Nrf2/ARE pathway through protecting Nrf2 protein from its degradation, resulting in the increasing Nrf2 protein and its nuclear translocation.

### 3.5. Induction of Antioxidant Enzymes in the Liver of PG-Dosed Mice

**Figure 6:**
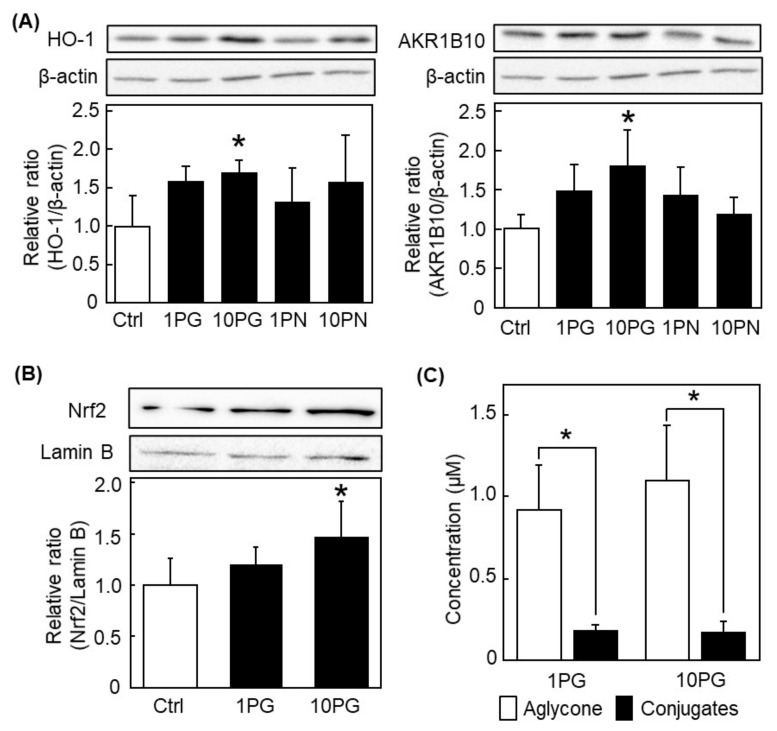
Induction of antioxidant enzymes in the liver of PG-administered mice and the concentration of incorporated PG in the plasma. Male ICR mice were orally administrated PG at 1.0 (1PG) or 10 mg/kg body weight (10PG) once a day for 1 week. The protein expression level of (**A**) HO-1 and AKR1B10 and (**B**) nuclear translocation of Nrf2 was determined by western blotting. Upper panels show typical blot data, while bottom ones are the intensity of specific bands after normalizing data by the β-actin (for (**A**)) and Lamin B (for (**B**)) levels. Values are the mean ± SE (*n* = 6), * *p* < 0.05 vs. control by Dunnett’s multiple comparison test. (**C**) The plasma concentrations of PG aglycon and conjugates were determined by HPLC. Values are the mean ± SE (*n* = 3), * *p* < 0.05 vs. control by student’s *t*-test.

To confirm the effect of PN and PG on the induction of antioxidant enzymes in vivo, these compounds were orally administered to male ICR mice and determined protein expression levels of HO-1 and AKR1B10 in the liver. PG at 10 mg/kg body weight significantly increased the protein expression of HO-1 and AKR1B10, but PN had no effect on the expression of these enzymes (Figure 6A). Furthermore, PG significantly promoted Nrf2 nucleus translocation (Figure 6B). These results indicated that PG could activate the Nrf2/ARE pathway and induced antioxidant enzymes in vivo as the same manner as the results from HepG2 cells.

To understand the relevancy of results between in vitro and in vivo experiments, the plasma concentrations of PG and its metabolite were measured after the consecutive administration of PG for 1 week at 1.0 and 10 mg/kg body weight once a day. Interestingly, the concentration of PG aglycone was significantly higher than that of conjugates in the plasma (Figure 6C). Concentration levels of aglycone in the plasma of PG-dosed mice were 0.92 ± 0.28 µM and 1.09 ± 0.34 µM, respectively, while that of conjugate forms were 0.18 ± 0.04 µM and 0.17 ± 0.06 µM, respectively. On the centrally, PN *per se* was not detected in the plasma of mice given PN for 1 week at 1.0 and 10 mg/kg body weight. However, the slight amounts of PG aglycone and its metabolites were detected in the plasma at 10 mg/kg body weight group: Concentrations of the aglycone and conjugate forms were 0.08 ± 0.11 µM and 0.23 ± 0.04 µM, respectively. These results suggested that PG mainly enter the body as the aglycone form and contribute to activate the Nrf2 pathway.

## 4. Discussion

In this manuscript, we demonstrated that PG had indirect antioxidant activity by inducing antioxidant enzymes, in addition to its direct antioxidant activity. PG enhanced the expression of antioxidant enzymes, such as HO-1, NQO1, and AKR1B10, in HepG2 cells (Figure 3). PG protected HepG2 cells from AAPH-induced oxidative stress (Figure 2C) through activating the Nrf2/ARE pathway (Figure 4 and Figure 5). Induction of antioxidant enzymes and its mechanism were confirmed in ICR mice (Figure 6A,B). This is the first report that PG can eliminate ROS indirectly by inducing antioxidant enzymes.

Polyphenols are known to possess antioxidant activity due to hydroxyl groups in their structures [3,4]. In particular, compounds with a catechol structure show potent antioxidant activity. PN and PG does not have this structure, but they have two hydroxy groups at 5- and 7-possition. These hydroxy groups would contribute to direct the antioxidant activity under cell-free conditions (Figure 1B). This direct antioxidant activity coincides with the previous studies that PN and PG exhibit DPPH radical scavenging activity [18,19]. Taken together, these results indicate that both PN and PG have direct antioxidant activity. In HepG2 cells, it was noteworthy that treatment with PG for 1 and 3 h to the cells failed to reveal antioxidant activity, but the antioxidant activity appeared 24 h after the treatment accompanied by the induction of antioxidant enzymes. Results from HPLC analysis showed that about 0.1% of PG was incorporated in HepG2 cells after 3 h-treatment but not in the cells after 24 h-treatment. These results suggest that the incorporated amount of PG after 3 h-treatment is not enough to reveal antioxidant activity, but it is of sufficient concentration to act as a trigger for the induction of intracellular antioxidant system and leads to appearance of the antioxidant activity after 24 h. On the other hand, PN had no direct antioxidant activity in HepG2 cells. PN may find it more difficult to permeate the cellular membrane than PG, because it has large molecular weight and hydrophilicity due to its rutinose moiety.

It was reported that certain polyphenols, such as luteolin, quercetin, and curcumin, possess indirect antioxidant activity through the induction of antioxidant enzymes [6,8,25]. In this study, we found that PG increased the protein expression level of HO-1, NQO1, and AKR1B10. HO-1 is known to metabolize heme into biliverdin/bilirubin, ferrous iron, and carbon monoxide, and it contributes to show the cytoprotective effects against various stress conditions [26]. For example, HO-1 is reported to possess hepatoprotective function from lipopolysaccharide-induced inflammation [27], ischemia-reperfusion injury, and fatty liver [28]. NQO-1 is a homodimer flavoprotein that catalyzes a two-electron reduction of electrophilic quinone substrates. The two-electron reaction prevents the quinone toxicity, because the reduction of quinones by one-electron reductases forms semiquinones, which generate ROS in the presence of molecular oxygen [29]. AKR1B10, a cytosolic member of the aldo-keto reductase superfamily, catalyzes the reduction of aldehydes such as 4-hydroxynon-2-enal and 4-methylpentanal [30]. Therefore, these enzymes play an important role in the protection of the liver from oxidative stress. It is known that expression of these antioxidant enzymes is regulated by an Nrf2/ARE pathway.

Nrf2 is a transcription factor that induces the phase 2 drug-metabolizing enzymes including antioxidant enzymes, by translocating into nucleus and binding to the ARE found in the promoter region of genes of various phase 2 enzymes [31]. Our findings revealed that PG increased protein expression (Figure 5A) and nuclear accumulation of Nrf2 (Figure 4A), a transcriptional activity of ARE (Figure 4B), and modification of Keap1 (Figure 5C). Keap1, which is a negative regulator of Nrf2, forms a homodimer via its BTB domain and binds to the Neh2 domain of Nrf2 with its DC domain [32]. Under normal conditions, Nrf2 receives ubiquitin-proteasomal degradation by Keap1 in cytoplasm. On the other hand, in the presence of ROS or electrophiles, the cysteine residues in Keap1 are covalently modified and lead to the conformational changes of Keap1, then Nrf2 escapes from its degradation [11,12]. Subsequently, Nrf2 translocates into the nucleus, binds to ARE, and starts transcription of Nrf2-regulated antioxidant enzyme genes [33], including HO-1, NQO-1, and AKR1B10. It has been reported that the activators of the Nrf2/ARE pathway react with the cysteine thiol group in the structure of Keap1. For example, *t*-BHQ [34] and sulforaphane [35] react with Cys151 of Keap1 and form a C-S bond. Certain polyphenols, such as luteolin [6] and quercetin [8], also induce modification of Keap1 and exhibit indirect antioxidant activity. Thus, PG is suggested to act in the same manner as these chemicals for activating the Nrf2/ARE pathway through promoting Keap1 modification.

In the present study, we confirmed that subsequent administration of PG for 1 week significantly increased the protein expression of HO-1 and AKR1B10 through promoting nuclear translocation of Nrf2 in the liver of mice (Figure 6A,B). To activate the Nrf2/ARE pathway, PG should enter the body. Indeed, the aglycone form of PG reached around 1.0 µM in the plasma (Figure 6C). This concentration of PG is relevant to that used in cell culture study. It should be explained why the concentration of aglycone was higher than that of conjugates in the plasma. As mentioned above, PG has two hydroxyl group in the structure at C5 and C7 positions. It is reported that C7-metabolite, but not C5-one, was found after PG administration in rats [36]. Conjugation at C5 position may be disturbed by the chemical groups in C4 and C6. Therefore, PG might mainly exist as the aglycone form in the plasma.

Excessive amount of ROS causes damage to the tissue and/or cells by interacting with cellular macromolecules, i.e., proteins, lipids, and DNA [37]. ROS-caused damage is associated with the onset of many diseases, such as liver diseases [37], cardiovascular disease [38], and diabetes mellitus [39]. Induction of antioxidant enzymes is recognized as one of the defense systems against oxidative stress. In this study, we demonstrated that PG induced antioxidant enzymes through activating the Nrf2/ARE pathway in HepG2 cells and the liver of mice for the first time. Thus, PG might be an attractive compound for the protection of liver from oxidative stress as a potential antioxidant through the induction of antioxidant enzymes.

## Data Availability

The data supporting the conclusions of this article are included within the manuscript.
